# Spatial and Machine Learning Approach to Model Childhood Stunting in Pakistan: Role of Socio-Economic and Environmental Factors

**DOI:** 10.3390/ijerph191710967

**Published:** 2022-09-02

**Authors:** Muhammad Usman, Katarzyna Kopczewska

**Affiliations:** Faculty of Economic Sciences, University of Warsaw, 00-927 Warszawa, Poland

**Keywords:** child malnutrition, climate vulnerability, multi-dimensional poverty, spatial regression, spatial clustering

## Abstract

This study presents the determinants of childhood stunting as the consequence of child malnutrition. We checked two groups of factors—the socio-economic situation and climate vulnerability—using disaggregated sub-regional data in the spatial context. Data related to the percentage of stunted children in Pakistan for 2017 were retrieved from MICS 2017-18 along with other features. We used three quantitative models: ordinary least squares regression (OLS) to examine the linear relationships among the selected features, spatial regression (SDEM) to identify and capture the spatial spillover effect, and the Extreme Gradient Boosting machine learning algorithm (XGBoost) to analyse the importance of spatial lag and generate predictions. The results showed a high degree of spatial clustering in childhood stunting at the sub-regional level. We found that a 1 percentage point (p.p.) increase in multi-dimensional poverty may translate into a 0.18 p.p. increase in childhood stunting. Furthermore, high climate vulnerability and common marriages before age 15 each exacerbated childhood stunting by another 1 p.p. On the contrary, high female literacy and their high exposure to mass media, together with low climate vulnerability, may reduce childhood stunting. Model diagnostics showed that the SDEM outperformed the OLS model, as AIC_OLS_ = 766 > AIC_SDEM_ = 760. Furthermore, XGBoost generated the most accurate predictions in comparison to OLS and SDEM, having the lowest root-mean-square error (RMSE).

## 1. Introduction

Child malnutrition is a form of undernutrition that results from nutrition-related deficiencies and dietary imbalances. It is further categorised into three anthropometric measures; i.e., stunting, wasting, and underweight [1]. Specifically, stunting refers to compromised growth and development when children fall two standard deviations below the expected height for their age. Stunting is largely determined by the child’s “first 1000 days”. During this phase, the child experiences its most rapid growth and development. It occurs predominantly due to long-lasting food and nutrient inefficiency [2]. During the last few decades, childhood stunting emerged as a major public health concern for low- and middle-income countries. Estimates from the Global Nutrition Report [3] imply that globally, the total number of stunted children will be 131 million by 2025.

There exist substantial region-wise disparities in childhood stunting. South Asia bears the maximum number of stunted children, followed by sub-Saharan Africa. UNICEF data showed that in 2018 in South Asia, 64 million children suffered from stunting [4]. Specifically, Pakistan, a South Asian country, has the third-highest childhood stunting rate, as high as 40.2%, after India and Nigeria. Approximately 12 million children less than five years of age were found to be stunted in Pakistan [5].

Stunting is considered to have adverse ramifications for child health and economic development. Horton [6] concluded that childhood stunting is linked to extensive health and economic consequences. Stunted children have higher mortality rates in comparison to non-stunted children. The poor health status of children increases the burden of diseases and thus increases poverty. It may impair their cognitive development and, consequently, their long-term earnings [7]. 

In Pakistan, previous studies examined key determinants of childhood stunting only from the socio-economic and demographic perspectives—they indicated poverty, poor sanitation and hygienic facilities, an increasing birth rate, and illiterate parents as the key contributing factors [8,9]. Furthermore, the spatial relations and spillover effects of both climate vulnerability and socio-economic factors on childhood stunting at the district level in Pakistan have not been explored yet [10]. Spatial models can capture the potential effects of neighbouring geographic units and the variability across geographic locations. The estimates of spatial models are essential in formulating and identifying context and location-based targeted interventions to reduce the effect of climate vulnerability and socio-economic factors on childhood stunting [11]. Furthermore, non-linear relationships and their related impacts have not been investigated thoroughly [12]. Therefore, this research aimed to address the following research question:

- Are there any spatial spillover effects of socio-economic factors and climate vulnerability on childhood stunting?

To answer this question, we ran an analysis containing the following steps:

- Examine the spatial distribution and clustering in childhood stunting at the district level in Pakistan;
- Jointly analyse the socio-economic and environmental factors of malnutrition;
- Examine whether childhood stunting is spatially dependent across districts;
- Compare and forecast district-level childhood stunting using OLS regression, spatial regression, and a machine learning algorithm to identify whether a structural change might be observed;
- Propose context and location-based policy recommendations.

The construction of this study is as follows. The next section provides an overview of childhood stunting and summarises the literature review. Section 3 demonstrates the research methodology: we used ordinary least squares (OLS) as a typical model; additionally, we used a spatial econometric model to capture spatial autocorrelation, neighbourhood clustering, and spillovers, and we applied the Extreme Gradient Boosting (XGBoost) machine learning (ML) model to validate the results with regard to possible non-linear relations and partial impact. Section 4 explains the results and discussion, and the last section provides conclusions and policy recommendations. This study brings novelty in the scope of the analysis (socio-economic and environmental factors of childhood stunting considered jointly), dimensions (spatial clustering and spillover), and methodological approach (OLS vs. spatial estimation and machine learning modelling). We show that this innovative combination made the study comprehensive and much more explanatory than other studies that only conducted partial analyses.

## 2. Literature Review on Child Malnutrition

### 2.1. Facts and Figures on Child Malnutrition

**Figure 1:**
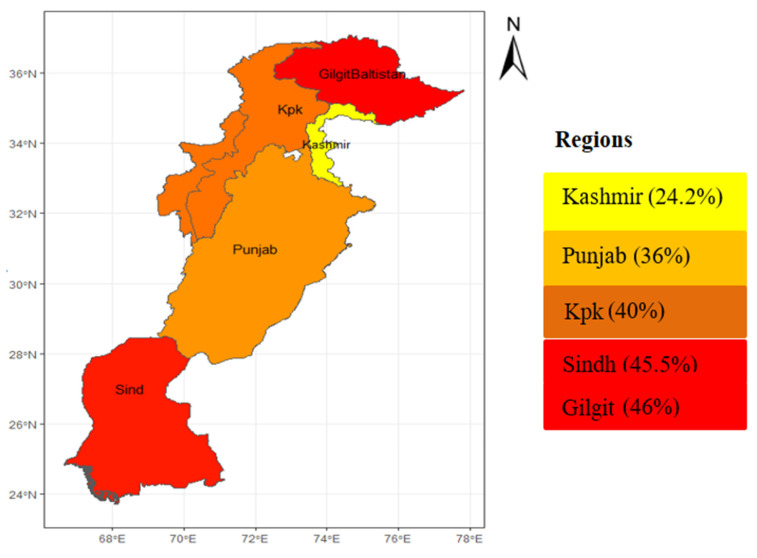
Regional spatial variation in childhood stunting in Pakistan 2017 (based on [5]).

In 2019, around 109 million fewer children throughout the world experienced stunted growth compared to 1990. Even if Pakistan made modest progress in reducing stunting rates, inter-regional disparities exist. Most stunting prevalence is concentrated in South Asia (56.1 million children, or 38%) and sub-Saharan Africa (57.5 million, or 41%) [13]. Explicitly, in 2013 Pakistan had the highest rate of childhood stunting in the South Asian region [14]. Figure 1 shows the regional spatial distribution of childhood stunting based on data from the Pakistan National Nutritional Survey of 2017. It suggests that the region of Gilgit Baltistan had the highest stunting percentage; i.e., 46%, followed by Sindh at 45.5%, Khyber Pakhtunkhwa (KPK) at 40%, Punjab at 36.4%, and Kashmir at 24.2%. Additionally, the national level estimate is at an alarming rate of 40.2%, posing a severe threat to child cognitive development and long-term growth [5]. The government of Pakistan was unable to achieve the 2017 Sustainable Development Goals (SDGs) target of reducing childhood stunting from 44% to 34%.

### 2.2. Determinants and Consequences of Child Malnutrition

There are few socio-economic determinants of child malnutrition. Poverty is considered one of its significant causes. Poor households lack enough financial resources to provide food and essential micronutrients to their children. This restrains their children’s early development, thus causing stunting [15,16]. A study by the International Food Policy Research Institute [17] revealed various factors that could significantly impact nutrition and consequently childhood stunting. Those factors were poverty, social protection programs, sanitation, water and hygiene, education, and climate change. Similarly, a study of child malnutrition in Northeast India [18] showed that access to safe water and sanitation facilities significantly reduced childhood stunting. The authors further asserted that children of literate households were found to be healthier than children belonging to illiterate families. Literate households avoid seeking health-related consultations from traditional health healers. A long-term 150-year study of malnutrition in Chile based on stunting rates from military registers [19] indicated that the problem decreased when health improved, energy consumption increased, poverty declined, and child labour was limited. 

Empirical studies confirmed this relation. A study of Ethiopia [20] revealed that 44.9% of children under five years of age were severely stunted. They revealed that mean household size, poor wealth status, child age, and diarrhea were significant factors that affected childhood stunting. Additionally, they found a linear relationship between the odds ratio of stunting and a large mean household size, which might inflate food competition among the family members. Therefore, lack and shortage of food might result in poor dietary intake, especially in children, thus leading to stunted growth. Similar findings were revealed for Indonesia, Pakistan, and India [21,22,23,24,25]. Poor socio-economic status, unavailability of adequate sanitation and hygienic facilities, parental education, incomplete vaccination, and lack of safe drinking water were the most important covariates that significantly affected childhood stunting. They recommended that governments in those countries must implement appropriate and integrated political and social policy measures to promote and initiate health-intervention programs, intensify nutritional access, and provide and promote personal hygienic habits. Consequently, these policies would generate and enhance economic resources there by improving child health.

To identify the associated individual and household risk factors of childhood stunting, the authors of [26] conducted a cross-sectional study in the North Mecha District, North West Region, Ethiopia. A detailed questionnaire was created to collect data. To identify the determinants of stunting, they used multilevel logistic regression. Furthermore, they used odds ratios to examine the degree of relationship between childhood stunting and independent features. A similar cross-sectional study design [27] classified predictors for stunting of children aged less than five years in Tanzania. They retrieved household, individual, and community-level data from the Demographic and Health Survey 2010. They used multi-level logistic regression to identify significant predictors. Their results suggested that children from poor households lacking basic sanitation and clean water facilities were found to be more stunted than children from non-poor households.

Recent literature linked child malnutrition with climate change. In the recent past, changing climatic conditions and their disproportionate negative consequences have intrigued many researchers to assess and quantify the impact of climate vulnerability on child health outcomes. It was reported that poor nutrition was the main contributing factor in nearly half of the deaths of children younger than five years. Moreover, this number is expected to grow because climatic shocks such as droughts, hurricanes, and floods are on the rise due to climate change [28]. A cross-sectional district-level study on India [29] assessed and identified the contextual correlates of childhood stunting: districts with extreme temperatures had high rates of childhood stunting. Crop production, wealth, and education had a negative effect on childhood stunting, manifesting that an increase in per capita crop production provided access to food. 

To further complement the association between climate vulnerability and child health, the authors of [30] estimated the health-related impacts of exposure to floods on children. They concluded that child malnourishment, especially stunting, was more likely to be exacerbated in the aftermath of the floods. Furthermore, they proposed that along with health and hygienic interventions, the government should introduce and implement climate-resilient and adaptative measures in regions more prone to climate change risks. Similarly, refs. [1,31,32] also tried to address climate change vulnerability and its implications on child health outcomes in India. The findings categorised 69 districts as being highly climate-vulnerable and bearing high rates of childhood stunting. These identified districts were also characterised by inadequate health and infrastructural facilities. The authors concluded that vulnerability to climate change intensified childhood stunting, meaning that the odds of suffering from childhood stunting increased by 32% if the district had been categorised as highly vulnerable compared to low-vulnerability districts. Furthermore, they found a significant spillover effect of high vulnerability on childhood stunting in the neighbouring districts. 

A spatial approach to analysing child malnutrition appeared slowly in the most recent literature. For example, refs. [1,2,33,34] examined the spatial autocorrelation between stunting and explanatory features for Zimbabwe, Ethiopia, Indonesia, and India, respectively. The authors generated maps to find spatial clusters and identified hotspots of stunting. To identify significant predictors, they used logistic regression. However, they did not employ spatial models to classify spillover effects on neighbouring districts, nor did they include any climatic or ecological factors. 

It was quite evident from the above literature that researchers attempted to quantify socio-economic, household, or climatic determinants separately [15,30]. A study that linked those two groups of factors appeared just recently, and but with a narrow scope [31]. Additionally, from a methodological perspective, we found that conventional econometrical techniques that are more suitable for linear data, such as ordinary least squares regression (OLS), still dominate the literature [22,23]. However, with the emergence of spatial models and machine learning techniques, it is apparent that these models are also growing in popularity due to their ability to deal with non-linear relationships [35]. We found quite a few studies that used such models. However, we observed that many studies were only based on spatial visualisation or cluster maps and did not clarify the importance and significance of spatial models and their components [22,36]. Therefore, this study attempted to address and fill those gaps by employing both modelling techniques—OLS and spatial models—to quantify the determinants of childhood stunting from socio-economic and climatic perspectives at the district level in Pakistan. We further used a machine learning model to validate the importance of spatial lag and calculated predictions based on assumed scenarios.

## 3. Research Design

**Figure 2:**
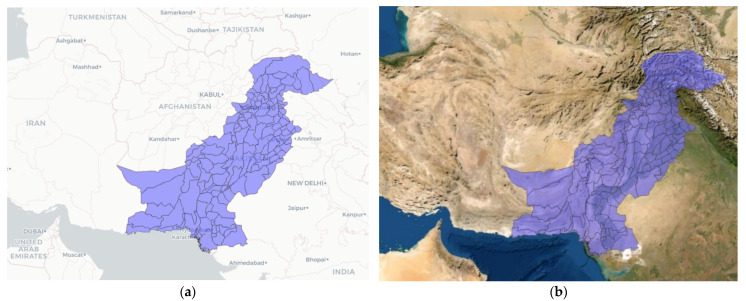
Regional map of Pakistan: (**a**) with administrative layer; (**b**) with topographic layer (both maps include all regions, including those that were not analysed due to a lack of data) * is location of capital city on the map.

Pakistan is divided into six regions, which further include 160 districts (Figure 2). Due to the unavailability of data for the province of Balochistan, this study used data for five regions comprising 116 districts. Regions were mapped using the shapefile of Pakistan from DIVA-GIS. All the processing and computation of data were conducted in R software (www.r-project.org (accessed on 15 November 2021)).

The major variable of interest (childhood stunting data) was taken from the 2017-18 Multiple Indicator Cluster Survey (MICS, https://mics.unicef.org/surveys (accessed on 15 November 2021)), which is a provincially representative data set for Pakistan. MICS is a cross-sectional survey that examined the district-level estimates of children (below five years of age) and women (aged 14–49 years) regarding health, population, nutrition, and other socio-economic indicators. The MICS sample frame followed the 2017 Pakistan National Population and Housing Census (PNPHC) as its sample design. It followed a standardised multi-stage, stratified clustering sample approach to select the survey sample for all regions. The first stage involved selecting primary sampling units (PSUs) comprising enumeration areas (EA). From each sampling strata, enumeration areas were drawn by using a probability proportional to the size-sampling protocols that solely depended on the number of households residing in each enumeration area at the time of the 2017 census. In the second stage, a systematic listing of households was carried out in all of the selected clusters by following an equal-probability systematic selection process. The survey teams only interviewed the pre-selected households. To prevent bias, no replacements of or changes to the pre-selected households were allowed at the implementation stages (MICS, 2017-18).

This study measured chronic child undernutrition according to stunting (height-for-age). Stunting, as a measurement of chronic undernutrition, affects the linear growth of a child, which reflects long-term nutrition deficiencies [37]. According to WHO standards and protocols, the MICS sample classified children as stunted if their height-for-age z scores fell below minus two standard deviations. Stunting was aggregated into percentages at the district level. For our analysis, we used the percentage of children below five years of age that were declared stunted for each district. 

Explanatory variables included two groups of features. The first group, socio-economic factors, included mean household size, multi-dimensional poverty, women’s exposure to mass media, female literacy rate, pre-mature birth, full immunisation, and marriage before age 15. All of the aforementioned independent features were retrieved from MICS (2017-18). All these features except mean household size were aggregated in percentage for each district (MICS, 2017-18). The second group, environmental features, included the climate vulnerability for each district. The data were retrieved from an integrated contextual analysis (ICA) [38] that classified the districts based on their exposure to climate change. The ICA comprised two core dimensions: vulnerability to natural hazards for floods and droughts. Each district was categorised as having low, medium, or high vulnerability depending on its vulnerability to climate change by keeping the exposure to the classifications mentioned above (ICA). All these features were in categorical form. Climate vulnerability was a composition of two main hazards: floods and droughts. Flood hazard index data, which were obtained from the National Disaster Management Authority (NDMA), represented a number of recorded flood events from 1950–2015. Drought data was obtained from the Pakistan Metrological Department and was based on soil moisture, precipitation, dependency on seasonal rainfall, and drought frequency (based on the Standardised Precipitation Index). All districts were classified on a 3-point scale as low, medium, and high based on their vulnerability to droughts and floods.

A spatial analysis of the problem was conducted using explanatory spatial data analysis (ESDA). We applied Moran’s *I* statistic to check if childhood stunting rates clustered in nearby districts. We analysed the geographical and spatial distribution of childhood stunting based on the Moran scatterplot and its mapping. The Moran’s *I* statistic is given by (1):

(1) $$I=\frac{N}{\Sigma_{i}\Sigma_{j}\omega_{ij}}\frac{\Sigma_{i}\Sigma_{j}\omega_{ij}\left(X_{i}-X\right)\left(X_{j}-X\right)}{\Sigma_{i}{\left(X_{i}-\bar{X}\right)}^{2}}$$

where *ω_ij_* represents the spatial weights matrix (we always used the row-standardised contiguity matrix) and reflects the neighbourhood of locations. It takes the value 1 if two locations *i* and *j* are adjacent and takes 0 for non-adjacent locations. *X_i_* and *X_j_* are the real observed values corresponding to locations *i* and *j*, whereas $\bar{X}$ signifies the mean value of variable *x*; *N* covers the total number of districts in the study area. The Moran’s *I* value ranges between −1 and +1. The value within the interval (0, 1] indicates a positive spatial autocorrelation, which means that regions are similar to their neighbours in a given feature. Conversely, the value within the interval [−1, 0) means a negative spatial autocorrelation and further indicates that regions with dissimilar and nonrelated feature values are distributed closely in space [39]. Furthermore, a Moran’s scatterplot was created that depicted four quadrants: hotspots (districts with high values for childhood stunting were next to each other), cold spots (districts with low values for childhood stunting were next to each other), and spatial outliers (districts with high values of childhood stunting and low values for neighbourhoods and vice versa) [39].

The study’s core was in understanding the relationship between childhood stunting and possible explanatory factors. We applied three approaches. (1) The classical ordinary least squares (OLS) method was used to test the global linear relations between childhood stunting and explanatory features. However, it neglected information from the neighbourhood; the OLS model was diagnosed using Moran’s *I* to determine if a spatial dependence existed in its residuals. A statistically significant Moran’s *I* value implies the need to develop a geo-spatial model that yields unbiased association estimates. (2) In the case of a significant Moran’s *I* value, we applied a spatial econometric model, which tested the linear relations and included information from the neighbourhood because the observations of childhood stunting were affected in the neighbourhood district as well. (3) We used the XGBoost machine learning model to test the non-linear relations and identify the most important features. All three approaches allowed for a high reliability of the results. All models tested the equation as follows:

(2) Stunting=f(poverty, houshold.size,immunization,marriage.before.15,female.literacy, premature.birth, mass.media.exp.women,flood.drought.low, flood.drought.high)

where stunting is the dependent variable, which meant the percentage of children who fell two standard deviations below the median height of age as declared by the WHO. The explanatory variables were: multi-dimensional poverty (MPI)—people of all ages living in poverty in all selected dimensions; the household size represented an average number of people in a household; immunisation showed the percentage of children aged between 12–23 months who received all basic vaccinations before the survey began; female literacy meant the percentage of women who attended secondary school and could read a short, simple statement; premature birth explained the percentage of children who born before 37 weeks of pregnancy; women’s exposure to mass media entailed the percentage of women who read a newspaper, listened to the radio, or watched television at least once per week; flood drought meant the degree of exposure to floods and droughts (low or high) (MICS, 2017-18; ICA, 2017). The multi-dimensional poverty (MPI) index consisted of three dimensions: *education, health,* and *standard of living*. *Health* and *education* had two indicators, while the *standard of living* had six indicators. All dimensions carried an equal weight of 1/3, while the weights of each component of the indicators differed. The *education* dimension included years of schooling and child attendance; the *health* dimension included nutrition and child mortality; and the *standard of living* dimension entailed electricity, sanitation, drinking water, housing, cooking fuel, and assets.

In the estimation process, we began with the OLS estimation. Then we computed the variance inflation factor (VIF) for all our explanatory features to examine the presence of multicollinearity. The VIF value for all the explanatory variables was below 5, suggesting that the OLS solution was stable. In the next step, we examined the spatial dependence in the error terms of the OLS residuals by using the Moran’s *I* value. A statistically significant value for Moran’s *I* justifies the applicability of spatial models—it confirms that residuals from the regression are similar in the neighbourhood (auto-correlated over space), and thus not random and independent and bearing some information. OLS estimates in the case of spatial dependency in its residuals are biased, inefficient, and inconsistent. We applied the spatial Durbin error model (SDEM), which controlled for spatial dependence in residuals and allowed for an assessment of the relationship between the dependent and explanatory variables with reference to the situation of explanatory variables in the neighbourhood regions. This allowed for tracking if the determinants of malnutrition in the neighbourhood mattered.

The machine learning XGBoost model checks the existence of relations in a non-linear setting. We cross-checked the significance of variables from the OLS and SDEM with the importance of variables from XGBoost. If the results of all three models were consistent, the linear models were a reliable tool in searching for determinants of the stunting rate. Variables that are non-significant in econometric models and highly important in machine learning (ML) models are linked to dependent variables in a non-linear way, which should be studied in detail.

**Figure 3:**
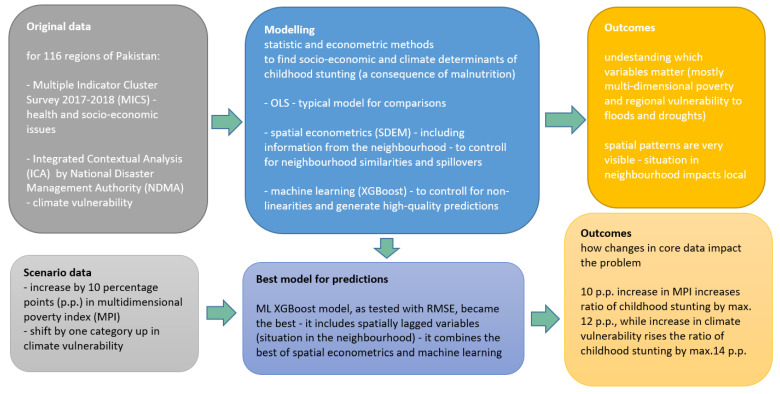
Flowchart of the study.

We summarised our study with the following flowchart (Figure 3). 

## 4. Empirical Results

**Figure 4:**
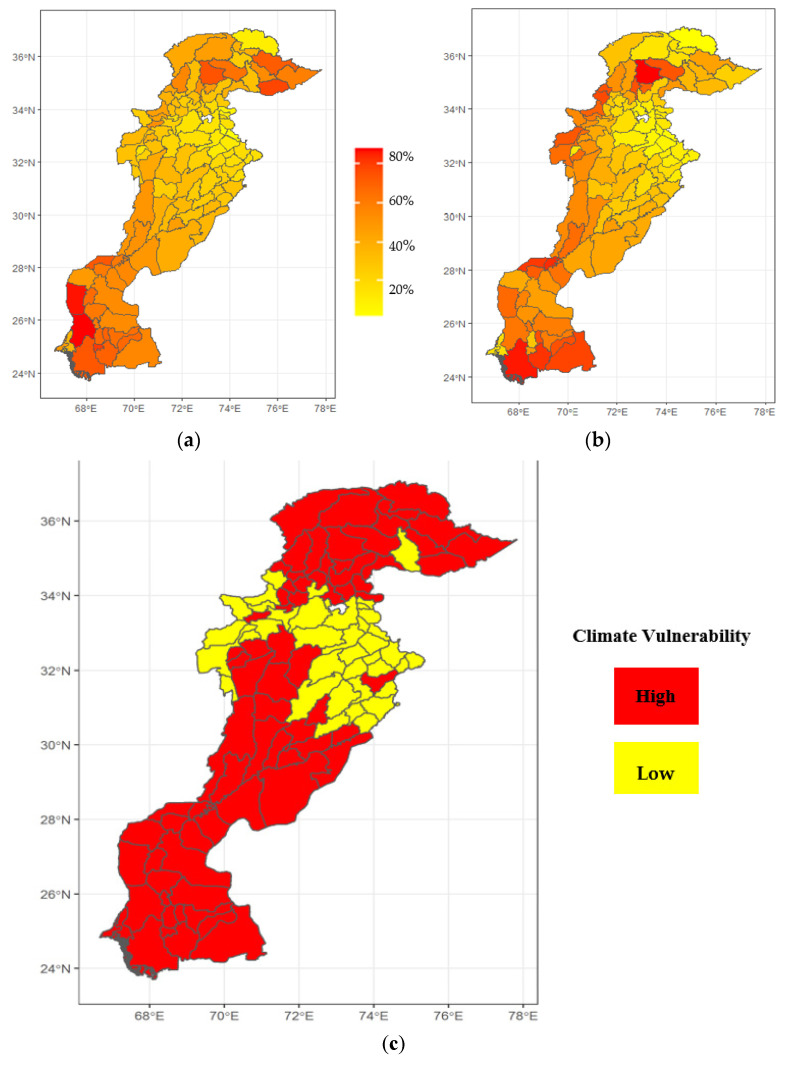
District-wise spatial distributions in Pakistan: (**a**) childhood stunting rate (%); (**b**) multi-dimensional poverty index (MPI) (%); (**c**) climate vulnerability based on MICS 2017-18 and ICA 2017.

The spatial diversity of childhood stunting and its socio-economic and environmental determinants were visible not only at the regional level, but also at the district level (Figure 4). Spatial maps revealed well-visible spatial patterns of agglomeration in the childhood stunting rate in Pakistan. Districts with low-to-moderate rates of childhood stunting were in the eastern part of Pakistan, specifically located in northern and central Punjab province (Attock, Gujrat, Gujranwala, Rawalpindi, Chakwal, and Jehlum) (see Figure 4a). Districts with high childhood stunting rates were widely distributed but mainly concentrated in the southeast (Punjab, Sindh), northwest (KPK), and northeast regions (Gilgit Baltistan). In general, districts belonging to those regions not only possessed higher stunting rates, but tended to be poorer (Figure 4b) and more vulnerable to climate change (Figure 4c).

The descriptive statistics presented in Table 1 show the scale of problems and their regional diversification among the regions of Pakistan. The average stunting rate in 2017-18 was 38.03%, regionally ranging from 13.6% to 72.3%. On average, the mean household size was 7–8 persons (7.5), the premature birth rate was 10.31%, and the full immunisation rate stood at 46.87%. Multi-dimensional poverty stood at 38.46%. Additionally, on average, 6.81% of females got married before reaching the age of 15 years, and less than half could read (41.91%).

**Figure 5:**
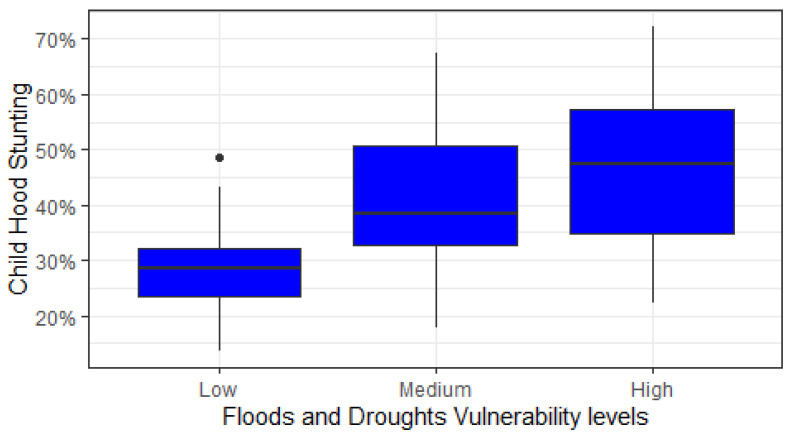
Box plots of regional childhood stunting rate (%) according to regions’ climate vulnerability—floods and droughts (low, medium, and high).

Figure 5 shows the childhood stunting rate in Pakistan according to different levels of climate vulnerability (floods and droughts): low, medium, and high. On average, regions highly vulnerable to floods and droughts report 58% of children were stunted, while in other areas, it was less—nearly 50% in medium-risk regions and only 35% in low-risk regions. These findings clearly suggested that we found a positive association between childhood stunting and climate issues: flood and drought vulnerability. 

The analysed variable; i.e., childhood stunting, was tested for spatial autocorrelation. Childhood stunting bore a significant positive autocorrelation (measured with a Moran’s *I* value) equal to 0.67 (*p*-value = 0.00010). These results confirmed that childhood stunting was not spatially independent, that visible spatial dependency in childhood stunting appeared, and the problem was similar in neighbouring regions. 

**Figure 6:**
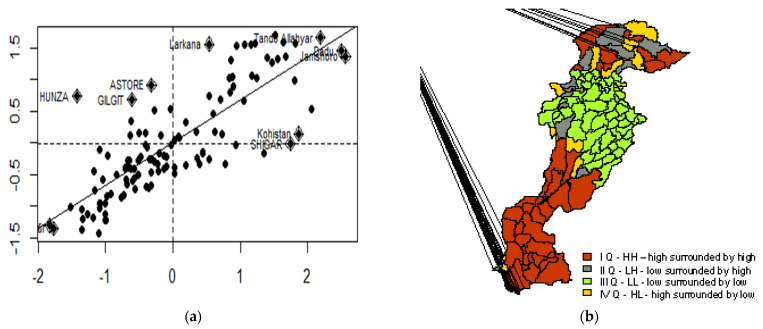
Childhood stunting in districts compared to the situation in their neighbouring regions: (**a**) Moran scatterplot; (**b**) mapped values of Moran scatterplot.

The incidence of spatial autocorrelation in childhood stunting was further examined using a Moran’s scatterplot (Figure 6a) and its mapping (Figure 6b). The Moran’s scatterplot (Figure 6a) illustrated values of regions (x) compared with average values in the neighbouring areas (y). This average neighbourhood value was calculated as the spatial lag, while information on which districts were neighbours was included in the spatial weights matrix (W). We applied the criterion of sharing a border to determine which districts were neighbours. Each district was compared to its neighbours; as the variable (childhood stunting) was standardised (mean = 0, st.dev = 1), its values reflected the regions’ relative position among all districts. The belongingness of points to given quarters of the Moran scatterplot (I, II, III, and IV) was mapped (Figure 6b)—the green colour signifies low-value regions surrounded by low-value regions (low–low, LL), while the red colour signifies high-value regions surrounded by high-value regions (high–high, HH). Figure 6b shows the clusters of low values (middle districts) and high values (southern districts).

Districts and their neighbouring regions with similarly low values were grouped in the 3rd quadrant as low–low regions (x < 0 and y < 0). In contrast, districts and their neighbouring regions with high values of childhood stunting were grouped in the 1st quadrant as high–high (x > 0 and y > 0). Additionally, the remaining districts with either high–low or low–high values represented the 2nd and 4th quadrants. They were named as spatial outliers or were regions lying on the border between areas with high and low levels. 

The estimation results are presented in Table 2. OLS assumes that linear relationships among variables and regions are independent of each other. The OLS estimates showed that multi-dimensional poverty, full immunisation, marriage before age 15, exposure to mass media, and flood and drought vulnerability (low and high) were significantly associated with childhood stunting. By keeping other factors constant, if multi-dimensional poverty increased by 1 percentage point (p.p.), it may translate into a rise of 0.32 p.p. in the stunting rate (*p*-value < 0.001). Furthermore, districts with high flood and drought vulnerability levels were significantly associated with an average higher rate of 0.3 p.p. in stunting (*p*-value < 0.1) as compared to medium flood and drought vulnerability (base category). On the contrary, low exposure to floods and droughts was significantly linked to an average decrease of −0.76 p.p. (*p*-value < 0.05) in the stunting rate compared to medium flood and drought vulnerability (*ceteris paribus*). In the region with 100,000 children under five years, with an average rate of stunting of 38% and moderate environmental vulnerability, an increase in risk of high environmental exposure (by 0.3 for the dummy variable) translated to 38.3% of stunting, which puts 300 more children at risk. 

The spatial model included inter-regional relations and dependencies. When considering spatial relations, SDEM showed similar findings to the OLS model but with one visible difference: for socio-economic features, multi-dimensional poverty, marriage before age 15, and women’s exposure to mass media were statistically significant. Their coefficient values were lower than their counter OLS estimates (which was typical when controlling for similarity in the neighbourhood). However, environmental factors (floods and droughts) showed a much stronger impact on childhood stunting than OLS estimates. This showed a more severe increase in stunting for districts belonging to highly vulnerable regions compared to those with medium vulnerability. The significant spatial effect (λ = 0.24, *p*-value < 0.1) demonstrated the spatial dependency in the error term. Additionally, significant spatial lags (average in neighbouring regions) of mass media exposure reduced stunting by −0.029 p.p. (*p*-value < 0.01), while high flood and drought vulnerability increased stunting by 1.11 p.p. (*p*-value < 0.01), which indicated the spatial spillover effects in the nearby districts. This suggested that the worsening of climatic conditions negatively influenced malnutrition not only in a given region, but also in neighbouring districts.

The model diagnostics showed that the SDEM outperformed the OLS model, as AIC_OLS_ = 766 > AIC_SDEM_ = 760. The spatial regression results suggested that we could not ignore the spatial spillover effect of the spatial lag variables; i.e., flood and drought vulnerability (high). In general, the SDEM had fewer significant variables, suggesting that spatial effects took over the impact of other selected variables.

**Figure 7:**
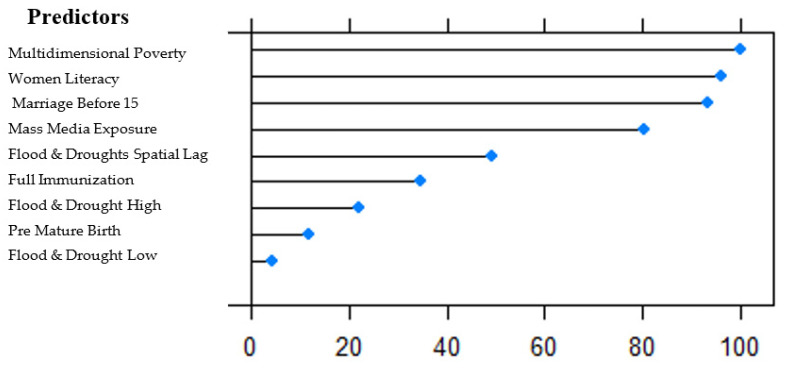
Variable importance as identified by XGBoost algorithm for predictions (the x-axis is in %; the y-axis lists the variables).

The next step was to retrieve the most important features as identified by the XGBoost algorithm. Figure 7 shows the most important predictors for childhood stunting. XGboost followed a two-step method in calculating feature importance. In the first step, it selected and ranked key variables. Then it selected the relative important indicators and found the ideal subclass of features using recursive feature elimination (picking more relevant features). XGBoost measured the relative importance of features in several ways, such as average gain or split weight method, etc. The most important features were used more frequently in building the model to obtain the final result, while the remaining ones are used to improve the residuals [40]. The most important feature was multi-dimensional poverty, followed by women’s literacy. Additionally, we observed that the spatial lag for floods and droughts was also quite important in predicting childhood stunting across neighbouring districts. It suggested that geographical boundaries did not limit the impacts of floods and droughts on childhood stunting, but extended them to nearby districts. We should note that the estimated ML model included a spatial component (spatial lags of floods and droughts); thus, it can be considered as a spatial machine learning model [41]. 

The last step of this study involved estimating predictions for the two assumed scenarios. The first case assumed a 10 p.p. increase in multi-dimensional poverty by controlling all other features. For the second case, we considered a 10 p.p. increase in vulnerability to floods and droughts by switching districts from low to high exposure and keeping all other features at the initial level. 

There are numerous evaluation measures to evaluate model accuracy in regression analysis, such as the mean absolute error (MAE), mean absolute percentage error (MAPE), root-mean-square error (RMSE), and mean squared error (MSE). We used the root-mean-square Error (RMSE) as the evaluation criterion to select the best model among OLS, SDEM, and XGBoost. The advantage of using RMSE over other measures is that it avoids the use of an absolute value. Furthermore, it is less affected by outliers in comparison to other evaluation measures, as squaring of errors will put a higher importance on outliers, which made our models unstable [42]. In short, RMSE informs about the standard deviation of residuals by identifying how much data is concentrated around the regression line. We calculated the RMSE for each model and compared their values. XGBoost turned out to be the most accurate model for our two assumed scenarios because it had the lowest root-mean-square error. 

**Figure 8:**
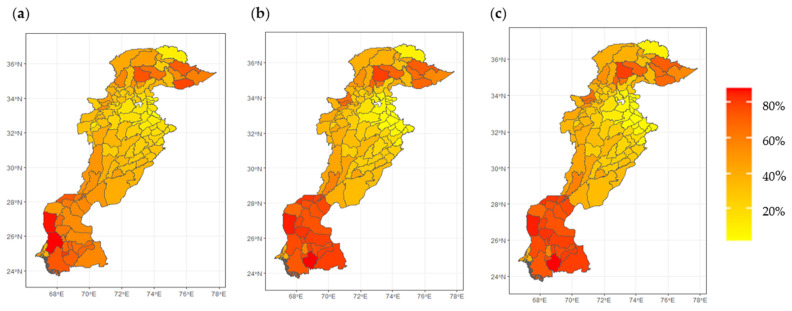
XGBoost predictions: (**a**) childhood stunting initial state; (**b**) result of 10 p.p. increase in MPI; (**c**) result of 10 p.p. increase in climate vulnerability.

In the case of the first scenario (10 p.p. increase in MPI), we observed an absolute rise in childhood stunting across all regions (Figure 8b). The most severe increase in childhood stunting was in the southeast region of Pakistan, specifically districts belonging to the Sindh province, where the changes reached 12 p.p. Table 3 shows the observed values, predicted values, and the final change observed in childhood stunting for the most affected regions.

In the second scenario (10% increase in climate vulnerability), districts in to the northeast region (Gilgit Baltistan) experienced increased childhood stunting (Figure 8c) with changes up to 14.2 p.p. Table 4 summarises the corresponding changes in real values of childhood stunting along with predictions in the northeast region. 

In both scenarios, the highest growth of stunting was predicted in the regions with the current best situation. This suggested that the malnutrition problems are highly instable and a slight worsening of situation may generate a strong response. From our estimated predictions, it seemed that high climate vulnerability worsened childhood stunting more than the increase in multi-dimensional poverty. One of the possible reasons was that floods and droughts tend to affect income-generating assets, which could lead to poverty. However, this does not mean the government should just focus on climate adaptation-related policies, as socio-economic vulnerability also plays a role in childhood stunting. Therefore, it is pertinent to tackle both issues by introducing and implementing contextual, integrated, and coordinated policies at the district level.

## 5. Discussion and Policy Recommendations

The findings of this research demonstrated that district-wise spatial distribution of childhood stunting in Pakistan is not random but is spatially correlated, and that districts are not independent. Out of the total of 116 districts, 27 locations had a childhood stunting rate above 50%. Districts with high childhood stunting rates were concentrated in the southern Punjab, eastern Sindh, northwest Khyber Pakhtunkhwa, and north Kashmir regions. On the other hand, low rates of childhood stunting were observed in the central and northern Punjab region. South Punjab and the eastern Sindh region of Pakistan were characterised by high multi-dimensional poverty, low women’s exposure to mass and media, a larger mean household size, and higher flood and drought vulnerability compared to more developed regions of the central Pakistan, northern Punjab, and Gilgit Baltistan regions. Remarkably, the geographical clustering of high childhood stunting rates coincided with the regions’ low socio-economic status. Our findings were highly consistent with the literature [21,28].

The presented study further explored the effects of climate vulnerability on childhood stunting. Notable findings showed very significant within-district spatial spillover effects of climate vulnerability on childhood stunting. Within the districts, there were significant effects of high climate vulnerability on childhood stunting regardless of the nearby locations. However, significant spatial spillover effects of climate vulnerability on childhood stunting deepened those relations and showed that geographical boundaries did not limit those effects. Our findings suggested that climate vulnerability is one of the major contributing factors to childhood stunting. Historical data indicate that due to abrupt climate changes, the number of extreme events such as floods and droughts has been on the rise in Pakistan. For example, devastating floods in 2010 affected more than 20 million people in the country. Additionally, 436 health care units and 80% of the food stocks were lost due to floods [43]. These findings can be related to the fact that such extreme events not only exacerbate food insecurity and cause food shortages, but may also affect child health, as more diseases are likely to emerge during such extreme events. Furthermore, children who are more likely to suffer from conditions such as diarrhea are more prone to suffer from child malnutrition and thus childhood stunting. 

The findings of this study asserted the need for more integrated, context-based, and targeted interventions to reduce childhood stunting in Pakistan. Socio-economic factors and climate vulnerability both had a significant impact on childhood stunting. Integrated and targeted strategies must be introduced to tackle economic empowerment along with climate vulnerability. The government must take into account multi-deficient locations and design strategies accordingly. For example, in regions such as eastern Sindh and northeast Gilgit Baltistan, which are multi-deficient, a mixture of social protection schemes and dietary-based programs for children should be implemented. Additionally, more efforts should be devoted to growing staple crops in such regions to resist climate vulnerability. Effective early-warning systems and informed land-planning measures should be introduced as adaptive policies. Our research identified geographical clusters based on locations that are known for multiple scarcities and deficiencies; it would be pertinent to introduce cluster-specific climate change adaptation and nutritional strategies depending on the needs of locations. 

## 6. Conclusions

This study presented critical findings for profound district-level policies for childhood stunting. The findings of this study showed the presence of district-wise spatial variation in childhood stunting in Pakistan. Geographical clustering and spatial patterns of selected indicators suggested a need to focus on and strengthen the district-level programs. This would further help in devoting limited resources to locations in high need. Climate vulnerability and socio-economic factors were identified as the major determinants of spatial variation of malnutrition effects. Our findings revealed that high climate vulnerability and poor socio-economic status worsened childhood stunting. Due to abrupt and drastic changes in climate, the entire population is at risk of being exposed to extreme events that could cause irreversible physical and intellectual damage. The present study further indicated that full immunisation, women’s exposure to mass media, and low climate vulnerability reduced childhood stunting. Therefore, it is critical to creating integrated, fact-based, contextual, and evidence-based policies to tackle the effects of climate vulnerability and poor socio-economic status on children. 

## Figures and Tables

**Table 1:** Descriptive statistics of analysed data for Pakistan 2017–2018.

Variables	Mean	Std.Dev	Min	Max
Stunting (%)	38.03	13.37	13.6	72.3
Mean household size (persons)	7.50	2.55	3.9	19.0
Pre-mature birth rate (%)	10.31	11.99	0.1	75.3
Full immunisation (%)	46.87	24.05	1.0	85.4
Multi-dimensional poverty (%)	38.46	23.15	2.0	87.8
Female literacy (%)	41.91	22.56	2.5	84.7
Women’s exposure to mass media (%)	37.97	30.28	0.0	86.6
Marriage before 15 (%)	6.81	4.26	1.4	24.1

**Table 2:** Estimated results from ordinary least squares (OLS) and spatial Durbin error model (SDEM).

Predicted Variables	OLS Model	SDEM Model
Multi-dimensional poverty	0.32 (0.06 ***)	0.18 (0.06 **)
Mean household size	0.10 (0.27)	0.11 (0.21)
Full immunisation	−0.19 (0.05 **)	−0.006 (0.04)
Marriage before age 15	0.83 (0.22 ***)	0.90 (0.19 *)
Female literacy	0.03 (0.07)	0.02 (0.08)
Pre-mature birth	0.03 (0.06)	0.04 (0.05)
Women’s exposure to mass media	−0.16 (0.04 ***)	−0.006 (0.06 *)
Flood and drought vulnerability (low)	−0.76 (0.79 *)	−1.44 (2.86)
Flood and drought vulnerability (high)	0.30 (0.89 *)	1.03 (3.96 *)
Spatial lag of mass media exposure Spatial lag of flood and drought vulnerability (high)		−0.29 (0.07 **) 11.1 (3.53 **)
Spatial lag λ	---	0.24 (0.09 *)
**Model Diagnostics**		
Adjusted R-squared AIC	0.71 766.99	0.83 760.48

Note: standard errors of regression estimates are in parenthesis; *p*-value levels: *** *p* < 0.001; ** *p* < 0.01; * *p* < 0.05; *p* < 0.1.

**Table 3:** Predictions and observed changes in case of the first scenario—10% growth in multi-dimensional poverty (most affected regions).

Districts	Initial Values	Predictions	Change
Tando Allahyar	67.3	79.3	12
Tando M. Khan	60.4	67.5	7.1
Jamshoro	72.3	78.7	6.4
Hyderabad	58.4	64.7	6.3
Jakobabad	62.1	68.2	6.1
Dadu	71.5	76.3	4.8
Nawab Shah	54.3	59	4.7
Umerkot	58.8	63.4	4.6

**Table 4:** Predictions and the observed change in case of the second scenario—10% growth in climate vulnerability (most affected regions).

Districts	Initial Values	Predictions	Change
Kharmang	63.5	77.7	14.2
Shigar	49.8	61.3	11.5
Ghanche	43.2	52.7	9.5
Gilgit	29.9	35.2	5.3
Ghizer	38.4	43.6	5.2
Skardu	48.4	52.3	3.9

## Data Availability

All data were collected from publicly open repositories and are available upon request.
